# Jugular Venous Compression via Soft Cervical Collar in Shunt-Refractory Low-Pressure Hydrocephalus: A Report of Two Cases

**DOI:** 10.7759/cureus.116100

**Published:** 2026-09-11

**Authors:** Yigal Bruk, Alexander Drofa, Evgueni Kouznetsov

**Affiliations:** 1 Department of Biology, Faculty of Science, York University, Toronto, CAN; 2 Neurological Surgery, Sanford Brain and Spine Institute, Fargo, USA

**Keywords:** cervical compression collar, jugular venous compression, low-pressure hydrocephalus, lph shunt failure, refractory ventriculomegaly intervention

## Abstract

Low-pressure hydrocephalus (LPH) is a rare and heterogeneous condition characterized by refractory ventriculomegaly despite low or negative intracranial pressure, often persisting after conventional CSF diversion. Established management strategies, including shunt revision and valve adjustment, frequently fail. We describe two patients, aged 45 and 66 years, in whom ventriculomegaly could not be controlled following ventriculoperitoneal shunting without continuous manual pumping of the shunt reservoir. As a result, bilateral jugular venous compression was applied using a soft cervical collar. Both demonstrated radiographic improvement, with third ventricular width decreasing from 13 to 8 mm and 13 to 4 mm in cases 1 and 2, respectively. Pumping was subsequently discontinued. In each patient, temporary cessation of collar use was temporally associated with recurrence of ventriculomegaly, which resolved following collar reapplication in case 1; the collar was not reapplied in case 2. Bilateral jugular venous compression with a soft cervical collar may represent a low-risk adjunct in shunt-refractory LPH. The response to withdrawal and reapplication observed here supports further investigation.

## Introduction

Low-pressure hydrocephalus (LPH) is a rare, clinically challenging, and underdiagnosed condition typified by persistent ventriculomegaly, refractory hydrocephalus, and clinical deterioration that fails to respond to CSF diversion despite low intracranial pressure (ICP) [1,2]. The rarity and paradoxical nature of this condition contribute to the frequency of misdiagnosis, with it commonly presenting as a sequela of subarachnoid hemorrhage (SAH) [3]. While its manifestation is not entirely understood, several proposed mechanisms have been discussed in the literature [1,4-7].

The predominant hypothesis, first introduced by Pang and Altschuler, focuses on the loss of viscoelasticity (turgor) in brain tissue or a gain in compliance [4]. This logic rests on the Monro-Kellie Doctrine, which states that the cranial cavity is a fixed-volume, rigid compartment consisting of CSF, blood, and brain tissue [8]. When one of the variables changes, the others must compensate; otherwise, ICP rises. In LPH, under the viscoelastic hypothesis, following hydrocephalus, parenchyma of higher compliance allows for ventricular expansion [9]; therefore, subsequent draining shows no reduction in ventricular caliber, as the brain tissue lacks the necessary elasticity to return to baseline. However, this mechanism remains unconfirmed.

A second hypothesis concerns loss of transmantle gradient between the ventricles and the sub-arachnoid space [6,9]. When the ventricles and subarachnoid space separate -- which can be caused by SAH or shear injury -- the subarachnoid space can develop very low pressure relative to the ventricles, thus causing them to expand [9]. Akins et al. favor an alternative mechanism, considering the brain as a highly permeable porous medium that, based on their poroelastic model, facilitates altered fluid dynamics in LPH [1]. These mechanisms are not mutually exclusive, and a combined model may better account for the observed phenotype.

Treatment options for LPH are as varied as the proposed mechanisms. The armamentarium includes sub-atmospheric (negative) pressure drainage, VP shunts, shunt revisions, neck wrapping, endoscopic third-ventriculostomy, and positional changes to the patient [2,3,5,9,10]. Drainage of any kind is highly invasive and risks infection, bleeding, misplacement, and catheter blockage [11]. When paired with the ambiguity of LPH pathogenesis, invasive shunt placements result in prolonged hospitalization, which can extend from several weeks to months [12]. Although sub-atmospheric drainage has historically been the preferred treatment option by clinicians, its protocol is not standardized, and drainage timelines are highly variable [13]. Patients with LPH frequently cannot be weaned to physiologic pressures, with ventricular re-enlargement and neurological decline on each attempt to raise the drain, contributing to the refractory nature of this condition and the prolonged hospitalization times.

Bilateral internal jugular compression reduces cerebral venous outflow and increases intracranial venous blood volume [4,10,14]. Under the Monro-Kellie doctrine, this added volume must be offset by displacement of another component [8]; where a functioning drainage pathway exists, CSF is removed rather than redistributed, and the parenchyma may re-expand at the expense of ventricular volume. The added venous volume is distributed within the parenchymal microvasculature, increasing tissue volume and interstitial pressure directly, without requiring elastic recoil of the mantle. We hypothesize that, with ventricular CSF drainage maintained, this may restore a transmantle pressure gradient sufficient to reduce ventricular caliber in the brain that had failed to re-expand with drainage alone.

This case series presents two patients in whom jugular venous compression using a soft cervical collar contributed to the management of refractory LPH. Compared to alternative interventions, bilateral jugular compression may provide a non-invasive, relatively low-risk therapeutic approach that can be applied at the bedside.

## Case presentation

Cervical collar protocol

In both cases, the indication for collar application was an inability to control ventricular size and hydrocephalus symptoms by any means other than continuous shunt valve pumping.

A standard soft foam cervical collar was used, with two small rolled towels placed bilaterally along the course of the internal jugular veins. The collar was tightened until a clearly visible indentation of the neck soft tissues appeared over the rolls, titrated to the maximum pressure the patient could tolerate. Indentation was confirmed visually after each adjustment, and patient responses were consistently observed. The goal was gentle jugular venous compression, avoiding carotid compression. The collar was worn continuously with brief interruptions for meals and hygiene.

Monitoring comprised serial neurological examination and interval CT to track ventricular caliber, with surveillance for discomfort, skin breakdown, dysphagia, dyspnea, facial or conjunctival venous congestion, and worsening headache.

The collar was discontinued only when two conditions were met together: neurological stability sustained for at least one week, and CT demonstrating improvement in ventricular caliber that was itself confirmed stable on subsequent imaging rather than on a single favorable scan. Reapplication was undertaken if ventricular caliber increased after discontinuation. This protocol was not pre-specified; it describes the technique as applied at the bedside and reconstructed from the clinical record.

Case 1

A 45-year-old woman with a history of hyperlipidemia and former tobacco use presented with progressive altered mental status following several days of poor oral intake and urinary incontinence. Non-contrast CT demonstrated diffuse SAH with intraventricular extension, hydrocephalus, and small subdural hematomas; CTA demonstrated fusiform dissecting aneurysmal dilation of both distal vertebral arteries.

On hospital day 2, she underwent right frontal external ventricular drain (EVD) placement for worsening hydrocephalus and bilateral vertebral artery flow diversion (pipeline embolization device, PED) on dual antiplatelet therapy (DAPT). Her course was complicated by stress-induced cardiomyopathy with severely reduced ejection fraction (subsequently improved) and delayed cerebral ischemia from severe multifocal vasospasm with basilar involvement. On hospital day 11, she deteriorated clinically and underwent emergent angiography with balloon angioplasty of the left V4 segment and mid-basilar artery and intra-arterial verapamil, with good angiographic response.

**Figure 1:**
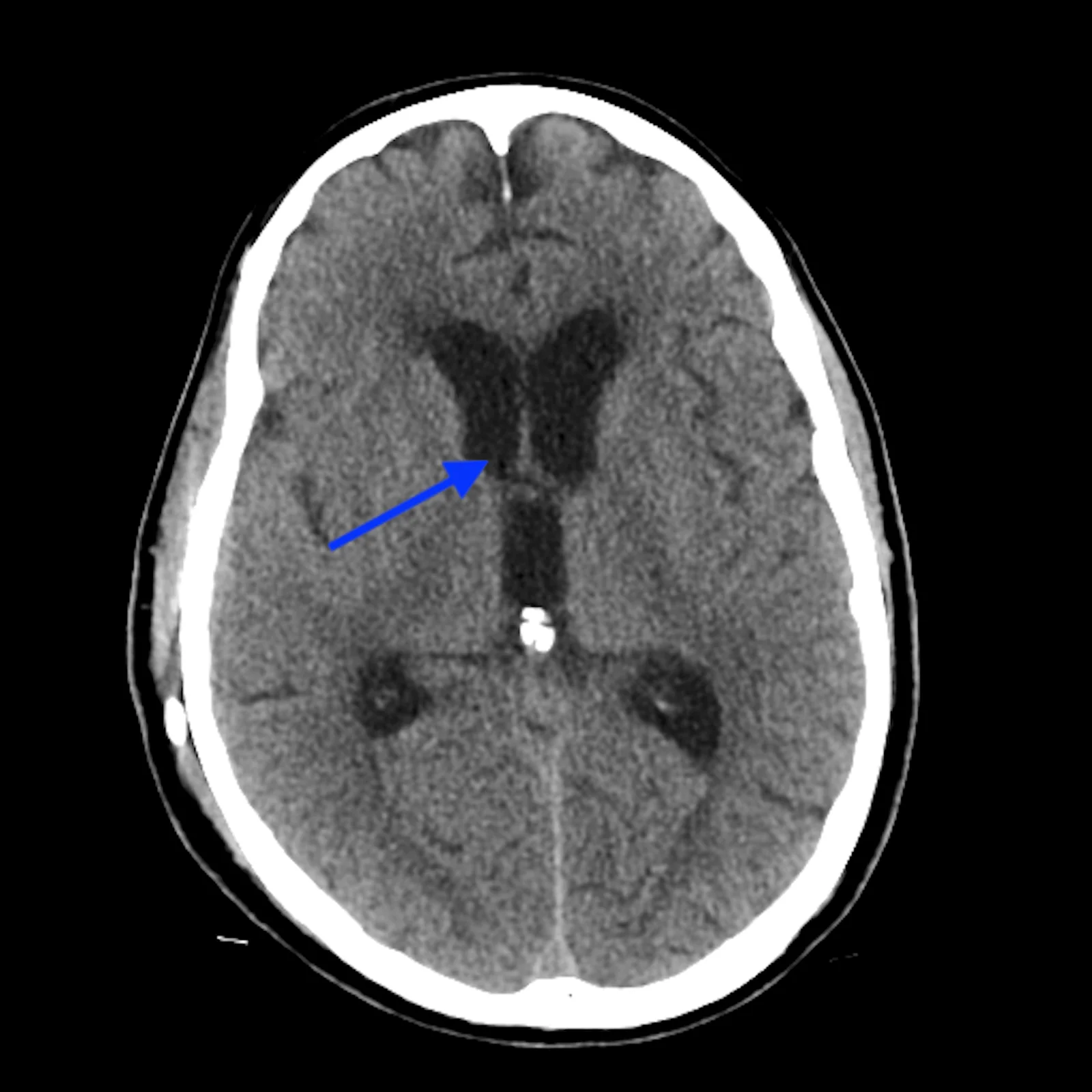
Axial non-contrast CT of the head following ventriculoperitoneal shunt insertion and before jugular venous compression, demonstrating persistent ventriculomegaly: third ventricular width 13 mm, temporal horns 6.2 mm (R) and 7.9 mm (L), and Evans ratio 0.30. Arrow indicates the right frontal horn. The ventricular catheter is not visualized on this section.

Repeated attempts to wean the EVD were unsuccessful. Measured ICP remained low at approximately 4 mmHg, yet continuous venting was required: while draining, the patient was alert with Glasgow Coma Scale (GCS) 15/15, whereas clamping produced drowsiness (GCS 13/15) and recurrent ventriculomegaly. On hospital day 18, a VP shunt was placed (programmable Strata valve, initial setting 1.0). Postoperatively, she continued to have episodes of somnolence with persistent ventriculomegaly (Figure 1), described in serial progress notes as obstructive hydrocephalus with low pressure. Clinical improvement was reproducibly observed only with manual pumping of the shunt reservoir, indicating inadequate effective drainage at the valve setting despite low ICP, a phenotype of LPH.

**Figure 2:**
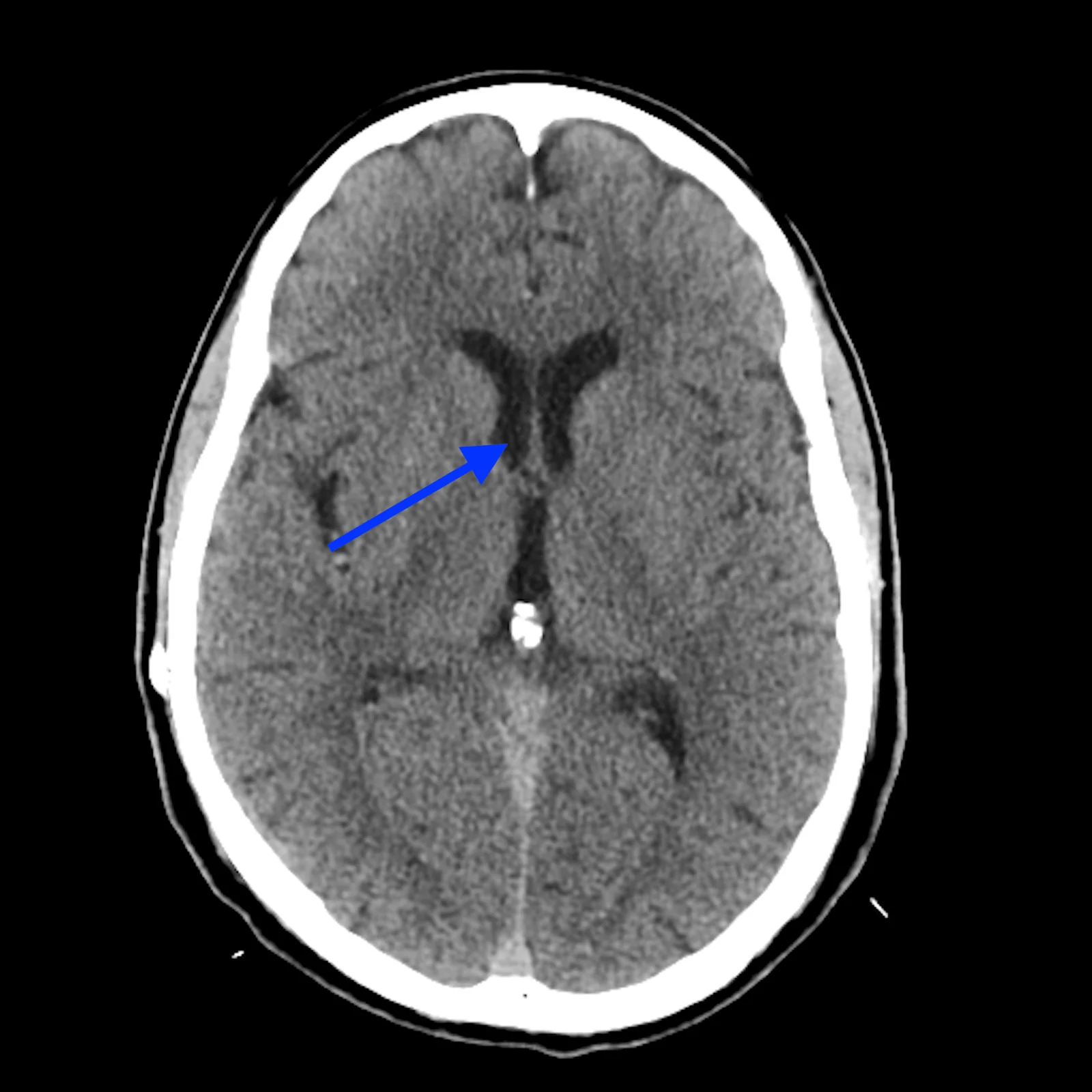
Axial non-contrast CT of the head following jugular venous compression with a soft cervical collar, demonstrating reduction in ventricular caliber: third ventricular width 8 mm, temporal horns 1.9 mm (R) and 2.1 mm (L), and Evans ratio 0.25. Arrow indicates the right frontal horn. The ventricular catheter is not visualized on this section.

Given that ongoing DAPT precluded further invasive intervention such as lumbar drain placement, a soft cervical collar was applied to provide light bilateral internal jugular compression. Alertness improved, ventricular caliber trended down, and manual pumping was discontinued. Subsequently, ventricular morphometry improved, with the Evans ratio falling from 0.30 to 0.25 and third ventricular width from 13 to 8 mm (Figures 1, 2). Measurements were obtained on comparable axial sections at the level of the foramen of Monro. GCS remained 15/15 off pumping. The collar was continued with allowance for short breaks and was tolerated without skin breakdown, dysphagia, dyspnea, or headache.

Twelve days after discharge to inpatient rehabilitation (hospital day 39), follow-up CT demonstrated interval recurrent ventriculomegaly, temporally associated with collar discontinuation during rehabilitation; the collar was reapplied and continued until outpatient reassessment. At outpatient follow-up approximately six weeks after discharge, she denied headache and reported continued collar use without adverse effect. She remained alert and oriented with no new focal deficits; residual binocular diplopia from bilateral abducens palsies persisted and was managed by neuro-ophthalmology with occlusion therapy. Interval CT demonstrated return to reduced ventricular caliber.

Case 2

A 66-year-old man was admitted with progressive communicating hydrocephalus 17 days after SAH from a basilar aneurysm treated with flow diversion (PED), maintained on DAPT with aspirin and ticagrelor. Non-contrast head CT on admission demonstrated interval enlargement of the ventricles, and repeat CT the following day confirmed persistent hydrocephalus with small-volume residual SAH and subdural blood products.

**Figure 3:**
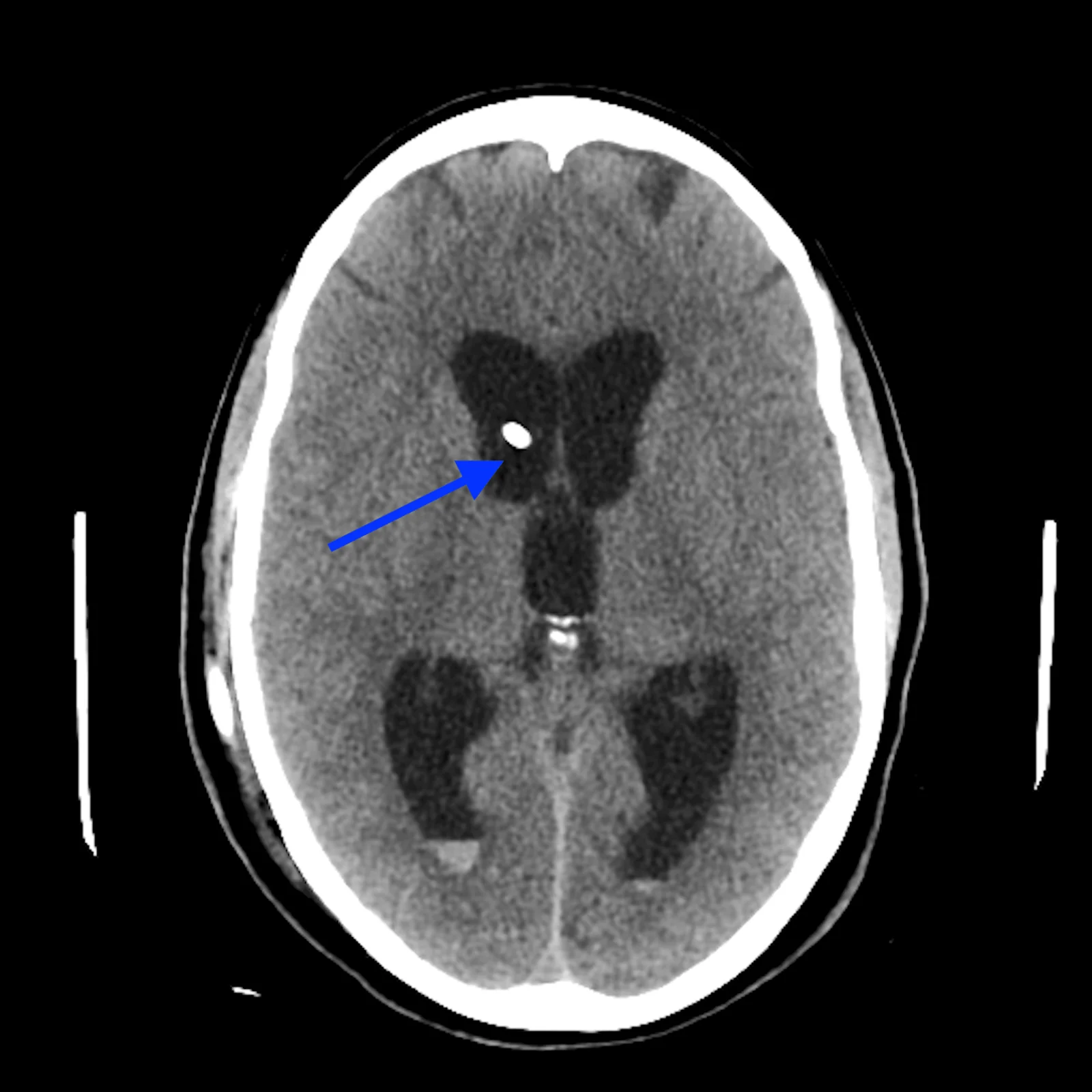
Axial non-contrast CT of the head following ventriculoperitoneal shunt insertion and before jugular venous compression, demonstrating persistent ventriculomegaly: third ventricular width 13 mm, temporal horns 10 mm (R) and 7.6 mm (L), and Evans ratio 0.34.

Given failure of conservative management and radiographic progression, he underwent VP shunt placement on hospital day 2 using a programmable Strata valve set initially at 0.5. Immediate postoperative imaging demonstrated expected postsurgical changes, including pneumocephalus and a small right lateral ventricular intraventricular hemorrhage, attributed to continued DAPT required to mitigate the risk of basilar stent thrombosis. An EVD was clamped for three days without any requirement for unclamping; measured ICP remained under 10 mmHg throughout, and level of consciousness was preserved, after which the drain was disconnected and removed. Serial CT demonstrated a fluctuating course with intervals of mild ventricular enlargement on hospital days 5 and 10, interposed periods of stability and gradual reduction on days 7 to 9 and 13 to 16, and stable but persistently enlarged ventricles by days 20 to 21 (Figure 3).

Neurologically, he improved and remained without focal deficits on serial examination. On hospital day 20, he was awake and conversant with an intact cranial nerve examination, full strength, and normal coordination, reporting only a brief episode of dizziness while ambulating. The following day, transient urinary incontinence and irritability were noted, although examination remained non-focal with preserved orientation and language. By hospital day 22, he was oriented with improving short-term memory and continued to report diplopia on left gaze, consistent with a prior sixth nerve palsy attributed to hydrocephalus at initial presentation. GCS was 15/15 throughout.

**Figure 4:**
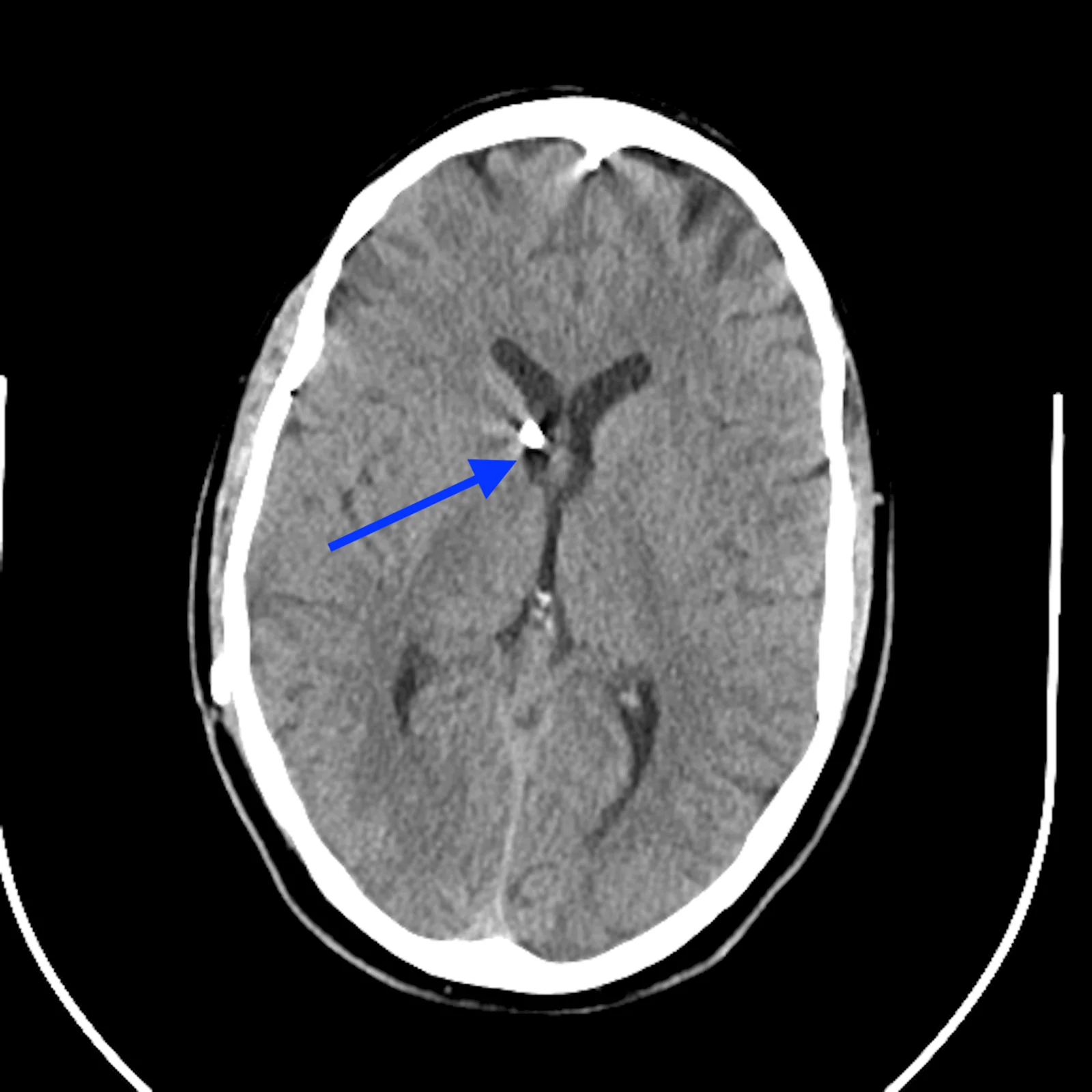
Axial non-contrast CT of the head following jugular venous compression with a soft cervical collar, demonstrating reduction in ventricular caliber: third ventricular width 4 mm, temporal horns 3.8 mm (R) and 3.8 mm (L), and Evans ratio 0.28.

Since ventricular size could not be stabilized other than by continuous shunt valve pumping, a strategy to augment intracranial venous pressure was instituted. The patient was permitted to lie flat while sleeping, and a soft cervical collar was applied for gentle bilateral internal jugular compression, worn continuously except during meals, with a cloth barrier to protect the skin. Ventricular morphometry improved after collar application, with the Evans ratio falling from 0.34 to 0.28 and third ventricular width from 13 to 4 mm (Figure 4); measurements were obtained on comparable axial sections at the level of the foramen of Monro. His examination remained stable, and serial imaging demonstrated stabilization of ventricular size, without skin breakdown, dysphagia, dyspnea, or headache.

After discharge, following discontinuation of jugular compression, follow-up CT demonstrated a slight increase in ventricular size, temporally associated with cessation of the collar. He remained clinically well, and surveillance imaging was arranged to document stability.

## Discussion

LPH remains a therapeutic challenge, typified by refractory ventricular enlargement alongside paradoxically low ICP [9]. Diagnostic frameworks commonly include hydrocephalus-related symptoms, low ICP, and an Evans index of >0.3 on CT [9,15]. The threshold defining the low-pressure state is variable across the literature. Hamilton and Price and Duan and Hu use <5 cmH2O [5,9], while Smalley et al. and Pang and Altschuler report ICP approximately below 10 mmHg [4,15]. The rarity of this condition is well documented; Czorlich et al. previously identified only 15 (3.7%) cases of acute LPH across 406 patients with aneurysmal SAH in a 10-year single-center series [16]. Standard shunt optimization is often inadequate, and salvage options such as lumbar drain placement carry a risk of hemorrhagic and other procedural complications [5,9,12]. In each of the cases presented here, DAPT was required to mitigate the risk of stent thrombosis, which precluded such surgical intervention. This constraint is what prompted a non-invasive adjunct, and it defines the clinical scenario in which cervical collar compression may be useful.

Jugular compression raises intracranial venous blood volume, which under the Monro-Kellie doctrine must be offset by displacement of another component [8]. Whether the resulting ventricular reduction reflects restoration of parenchymal turgor or displacement alone remains unresolved, and the available physiological evidence bears on that distinction directly. The soft cervical collar approach may function by a mechanism similar to that of sub-atmospheric drainage, where both enable ventricular shrinkage through alterations in transmantle pressure gradients, although turgor-related hypotheses cannot be excluded [6,10]. Importantly, clinical improvement has been reported to correlate with restoration of ventricular size, not ICP values [4], consistent with geometry rather than absolute pressure being the operative variable. The mechanism by which a soft cervical collar might reduce ventriculomegaly, or whether it does so at all, was not directly assessed in this case series.

Neck wrapping has been previously seen in the literature as a non-invasive armamentarium for LPH [3,5,9,10]; however, it has been sparsely examined as a discrete intervention. Physiological evidence remains mixed. One study found a significant reduction in jugular venous flow following bilateral compression in healthy volunteers (572.2 ± 219.7 to 460.8 ± 202.4 mL/min, p=0.008), while mean arterial inflow remained unchanged [10]. Subsequent magnetic resonance elastography demonstrated no significant difference in ∆G′ and ∆G″ -- the shear storage and loss moduli -- contradicting the hypothesis of a re-turgoring of brain tissue and lending support to a volume-displacement interpretation [10]. It is important to note that these measurements were conducted in healthy volunteers rather than individuals with LPH, and that an indwelling diversion device was not stated to be present in participants. Hamilton and Price report the use of neck wrapping or a neck tourniquet in 20 patients with an EVD at standard and sub-atmospheric pressures, proposing that elevated dural sinus pressure inhibits CSF drainage from the cortical subarachnoid space, allowing the mantle to re-expand [5]. Wrapping technique, compression points, and degree of compression were not specified, and because wrapping and sub-atmospheric drainage were applied together or interchangeably, the contribution of compression alone could not be isolated [5]. Notably, withdrawal after three days produced a decreased level of consciousness and recurrent ventriculomegaly in one illustrative case, whereas discontinuation of both the wrap and EVD after 10 days was followed by normal ventricular size in another [5]. These findings parallel the temporality of collar application to ventriculomegaly observed in our cases. Additionally, Hatt et al. reported that participants in whom venous outflow remained within the internal jugular veins during compression demonstrated higher brain stiffness than those exhibiting compensatory redistribution to extrajugular pathways [10]. On that point, Fargen et al. describe extrajugular collaterals dilating with chronic use in patients with longstanding jugular outflow impairment [14]; whether sustained collar compression induces comparable collateral recruitment in patients with LPH, and therefore attenuates its own effect over time, is unknown.

Building on the concept of hysteresis proposed by Lesniak et al., a modeled venous sinus thrombosis in a pediatric population produced persistently elevated compliance and ventricular enlargement at sub-atmospheric ICP, with compliance declining only once sub-atmospheric drainage was applied [7,17]. This supports venous pressure as a determinant of the LPH state based on the compliance (viscoelastic) hypothesis. However, it is a theoretical model, and pathological thrombosis differs from graded external compression in magnitude, reversibility, and thrombotic risk. It therefore cannot be taken as evidence for the collar itself. Prospective evaluation has been attempted; a clinical study designed to evaluate bilateral internal jugular compression via a Q-collar mechanism was withdrawn due to insufficient enrollment, leaving the evidence base reliant on case-level reports [18].

Given only two uncontrolled cases, a causal effect cannot be established, and several alternative explanations warrant consideration. Both patients were recovering from aneurysmal SAH, and gradual resolution of hemorrhage-related impairment of CSF absorption may account for delayed ventricular improvement independent of any intervention. In case 2, the patient was already improving neurologically and radiographically before collar application; therefore, a late response to standard shunting cannot be excluded. Moreover, this patient was permitted to lie flat concurrently with collar application, and positional change independently raises ICP and cerebral venous pressure [9,19], meaning the observed improvement in this case cannot be attributed to jugular compression alone. In case 1, manual valve pumping continued into the period immediately preceding collar application, further confounding attribution. Ventricular measurements were obtained from clinically indicated scans rather than a standardized imaging protocol, and minor differences in section level influence temporal horn measurement in particular. Importantly, the observation most resistant to these explanations is the sequence observed in case 1, where ventricular caliber decreased following collar application, increased after discontinuation, and decreased again upon reapplication; a single monotonic recovery does not readily produce such a pattern. In case 2, the patient demonstrated ventricular enlargement following discontinuation, but the collar was not reapplied.

A distinguishing feature of our approach is the use of a soft cervical collar with a skin barrier, which provides reproducible, gentle internal jugular compression while reducing the skin injury risk associated with tighter neck wrapping. The application differed between cases: a standard-orientation collar with intermittent breaks in the first case, and a reverse-worn collar worn near-continuously in the second. This reflects adaptation to patient anatomy and tolerance rather than a fixed protocol. Soft cervical collar compression is a non-invasive armamentarium that may provide clinicians with a comparatively low-risk strategy; nevertheless, the technique deliberately raises ICP and is not benign. Excessive or prolonged venous restriction may theoretically increase risk of venous congestion, cerebral edema, impaired cerebral perfusion pressure, or thrombotic complications, particularly in patients with pre-existing vascular pathology [14,20]. Serial neurological examinations and serial imaging to track ventricular caliber should be paired with surveillance for patient discomfort, skin breakdown, dysphagia, dyspnea, venous congestion, and headaches. Neither patient in this series experienced such complications; however, two patients cannot establish the safety of a treatment protocol.

## Conclusions

Both patients presented with hydrocephalus following aneurysmal SAH, with neurological deficits and urinary incontinence documented over the course of admission. Physiological anchors of LPH were identified, with refractory ventriculomegaly despite optimized shunt placements and low ICP. CT demonstrated recurrent ventricular enlargement following soft cervical collar cessation in both cases, with subsequent resolution following collar reapplication in the first. This temporal association may suggest a physiological relationship between jugular compression and ventricular size. However, confounders, including natural disease course, concurrent positional measures, and continued shunt pumping, cannot be excluded.

These two cases describe soft cervical collar compression as a non-invasive, relatively low-risk approach that deserves formal evaluation in patients with LPH. Our findings are broadly consistent with existing literature on cervical compression-based techniques. Given that LPH is often identified belatedly, the role of soft collar compression in the early clinical course remains poorly documented. The ventricular response to collar application and discontinuation may substantiate a physiologic effect, although these observations are hypothesis-generating rather than confirmatory. Further study is warranted to establish optimal candidate selection, compression parameters, duration, and weaning protocols.

## References

[1] Akins PT, Guppy KH, Axelrod YV, Chakrabarti I, Silverthorn J, Williams AR (2011). The genesis of low pressure hydrocephalus. Neurocrit Care.

[2] Kuppler P, Ditz C, Küchler J, Fieseler K, Kehler U, Leppert J (2025). Chronic negative-pressure hydrocephalus: management of prolonged subatmospheric drainage failure and treatment approach in persistent ventriculomegaly. Illustrative case. J Neurosurg Case Lessons.

[3] Keough MB, Isaacs AM, Urbaneja G, Dronyk J, Lapointe AP, Hamilton MG (2021). Acute low-pressure hydrocephalus: a case series and systematic review of 195 patients. J Neurosurg.

[4] Pang D, Altschuler E (1994). Low-pressure hydrocephalic state and viscoelastic alterations in the brain. Neurosurgery.

[5] Hamilton MG, Price AV (2012). Syndrome of inappropriately low-pressure acute hydrocephalus (SILPAH). Hydrocephalus.

[6] Ly-Liu D, Benatar-Haserfaty J, Bellver LS (2015). Low-pressure hydrocephalus: a case report. Int J Clin Anesthesiol.

[7] Lesniak MS, Clatterbuck RE, Rigamonti D, Williams MA (2002). Low pressure hydrocephalus and ventriculomegaly: hysteresis, non-linear dynamics, and the benefits of CSF diversion. Br J Neurosurg.

[8] Mokri B (2001). The Monro-Kellie hypothesis: applications in CSF volume depletion. Neurology.

[9] Duan S, Hu J (2024). Pathogenesis and management of low-pressure hydrocephalus: a narrative review. J Neurol Sci.

[10] Hatt A, Cheng S, Tan K, Sinkus R, Bilston LE (2015). MR elastography can be used to measure brain stiffness changes as a result of altered cranial venous drainage during jugular compression. Am J Neuroradiol.

[11] Zhou J, Zhong Y, Li X, Li H, Wang J, Yang S, Chen G (2023). Risk factors for external ventricular drainage-related infection: a systematic review and meta-analysis. Neurol Clin Pract.

[12] Godoy Hurtado A, Barstchi P, Brea Salvago JF, Al-Ghanem R, Galicia Bulnes JM, El Rubaidi O (2023). Low- and negative-pressure hydrocephalus: new report of six cases and literature review. J Clin Med.

[13] Bakmeedeniya RW, Hedger JS, Purnell MR (2026). Management of low/negative pressure hydrocephalus: a systematic review of etiology, pathophysiology, and treatment. World Neurosurg.

[14] Fargen KM, Midtlien JP, Margraf CR, Hui FK (2024). Idiopathic intracranial hypertension pathogenesis: the jugular hypothesis. Interv Neuroradiol.

[15] Smalley ZS, Venable GT, Einhaus S, Klimo P Jr (2017). Low-pressure hydrocephalus in children: a case series and review of the literature. Neurosurgery.

[16] Czorlich P, Schweingruber N, Göttsche J, Mader MM, Westphal M (2023). Acute low-pressure hydrocephalus in aneurysmal subarachnoid hemorrhage. Neurosurg Focus.

[17] Datta D, Tu A (2025). Pediatric low-pressure hydrocephalus: a case series and re-analysis of a rare disease. Childs Nerv Syst.

[18] ClinicalTrials.gov [Internet] (2026). The use of Q-collar to increase CSF drainage in low-pressure hydrocephalus patients. https://clinicaltrials.gov/study/NCT06129565.

[19] Alperin N, Lee SH, Sivaramakrishnan A, Hushek SG (2005). Quantifying the effect of posture on intracranial physiology in humans by MRI flow studies. J Magn Reson Imaging.

[20] Wang J, Manaenko A, Hu Q, Zhang X (2024). Cerebral venous impairment and cerebral venous sinus thrombosis. Brain Hemorrhages.

